# Debridement, Antibiotics and Implant Retention for Hip Periprosthetic Joint Infection: Analysis of Implant Survival after Cure of Infection

**DOI:** 10.7150/jbji.40924

**Published:** 2020-02-10

**Authors:** Martin Clauss, Christof Hunkeler, Isabella Manzoni, Parham Sendi

**Affiliations:** 1 Interdisciplinary Septic Surgical Unit, Clinic for Orthopedic and Trauma Surgery, Kantonsspital Baselland, Liestal, Switzerland; 2 Center for Muscular-Skeletal Infections, Department of Orthopedics and Traumatology, University Hospital Basel, University of Basel, Basel, Switzerland; 3 Medical Faculty, University of Basel, Basel, Switzerland; 4 Department of Infectious Diseases and Hospital Epidemiology, University Hospital Basel, University Basel, Basel, Switzerland; 5 Institute for Infectious Diseases, University of Bern, Bern, Switzerland

## Abstract

**Background**: Debridement, antibiotics and implant retention (DAIR) is a valuable option for treating early and acute periprosthetic joint infection (PJI). The inflammation caused by the infection and the surgical intervention during DAIR may influence the long-term stability of the implant. In this study, we analyzed the sequelae of DAIR on implant survival in hip PJI after cure of infection.

**Methods**: Total hip arthroplasties (THAs) from our database implanted between 1992 and 2016 were included in a retrospective double-cohort study. THAs were exposed (DAIR cohort) or not exposed to DAIR (control cohort). The control cohort comprised patients matched 3:1 to the DAIR cohort. The outcome was implant failure over time. It was evaluated for (i) revision for any reason, (ii) aseptic loosening of any component, and (iii) radiographic evidence of loosening.

**Results**: 57 THAs (56 patients) were included in the DAIR cohort and 170 THAs (168 patients) in the control cohort. The mean follow-up periods in the DAIR and control cohorts were 6.1 and 7.8 years, respectively. During follow-up, 20 (36%) patients in the DAIR cohort and 54 (32%) in the control cohort died after a mean of 4.1 and 7.2 years, respectively. Revision for any reason was performed in 9 (16%) THAs in the DAIR cohort and in 10 (6%) THAs (p=0.03) in the control cohort, and revision for aseptic loosening of any component in 5 (9%) and 8 (5%) THAs (p=0.32), respectively. Radiological analysis included 56 THAs in the DAIR cohort and 168 THAs in the control cohort. Two (4%) stems and 2 (4%) cups in the DAIR cohort and 7 (4%) and 1 (0.6%) in the control cohort, respectively, demonstrated radiological signs of failure (p=1).

**Conclusions**: THAs exposed to DAIR were revised for any reason more frequently than were THAs in the control cohort. The difference was mainly caused by septic failures. After cure of PJI, the difference in revisions for aseptic loosening was not significant. There was no significant difference in radiographic evidence of loosening of any component between cohorts. These data suggest that cured hip PJI previously exposed to DAIR do not fail more frequently for aseptic reasons than do THAs not exposed to DAIR.

## Introduction

The number of arthroplasty procedures being performed is increasing over time. It is estimated that, by 2030, more than 570,000 primary total hip arthroplasties (THAs) will be performed in the United States annually 1. The estimated rate of periprosthetic joint infection (PJI) of the hip is approximately 1% 2. Consequently, the absolute numbers of PJIs will increase in parallel to the growing number of THAs performed.

Zimmerli et al. 3 and the Infectious Diseases Society of America 4 published a treatment algorithm for the management of PJI. It consists of debridement, antibiotics and implant retention (DAIR) or 1- or 2-stage exchange of the arthroplasty as curative surgical strategies to preserve a functional joint. PJI within 30 days of prosthesis implantation or acute PJI (i.e., <3 weeks of symptoms) in the setting of a well-fixed prosthesis without a sinus tract can be successfully managed with DAIR 5.

In studies that investigate the outcome of PJI treatment ≥2 years after surgery, success is commonly defined as infection-free survival with a mobile joint. While long-term analysis of primary and revision THAs in non-infected cases have demonstrated results on the survival of cemented and non-cemented implant components 6-8, little is known on the sequelae of implant survivorship *after* successful DAIR. In other words, data are lacking on the long-term implant survival of hip arthroplasty components in patients who have been successfully treated with DAIR for a PJI.

The objective of the present study was to compare implant survivorship results of THAs exposed to the DAIR procedure and after presumably cure of infection with the results of THAs not exposed to this type of surgical intervention. We hypothesized that the long-term implant survival rate of THAs exposed to DAIR (and cure of PJI) would be lower than that of THAs not exposed to DAIR.

## Patients and Methods

### Study design, setting, and participants

All patients were identified from our prospectively collected hip arthroplasty database. The study period for THA inclusion was 24 years (1992 to 2016) and the database in the study period consisted of 5340 THAs. We used a retrospective double-cohort study design, nested in our prospective arthroplasty register. The study design is illustrated in the Supplementary Material (**Figure S1**). The denominator was THAs for all variables, except for age and gender.

*DAIR cohort*: The cohort consisted of primary and revision THAs exposed to DAIR with a curative intention because of PJI (designated as the DAIR cohort). The reasons for implant exchange in included revision THAs was not evaluated. The data related to the DAIR cohort have been used in part and in a different context in a previous study 5. The definitions used for the diagnosis of PJI and the surgical technique for DAIR at our institution are described elsewhere 5, 9. In brief, clinical signs for PJI included pain, warmth, erythema, induration, and sinus tract. The diagnosis of PJI included clinical signs plus at least one of the following criteria: (1) growth of the same microorganism in at least two cultures of synovial fluid and/or periprosthetic tissue, (2) visible pus surrounding the joint, (3) acute inflammation on histopathologic examination (>10 neutrophils/high-power field). No intra-operative rapid tests (e.g., alpha defensin test, frozen section for histology) were used. The antimicrobial treatment duration for PJI with curative intention was 3 months 5, 9. Then, patients were followed for implant survival.

*Control cohort:* The second cohort for comparison consisted of primary or revision THAs not exposed to DAIRs (designated as the control cohort). The reasons for implant exchange in revision THAs was not evaluated. However, at the time of study inclusion (Supplementary Material **Figure S1**), all subjects were considered to be infection free. Thus, primary THAs in the control cohort were never exposed to DAIR and had never experienced an infection.

### Follow-up examinations within cohorts

In both cohorts, THAs were followed for stability. The study period for follow-up examination consisted of 26 years (1992 to 2018, 2 years after the last included patient). Follow-up examinations were scheduled at 4 months, 1 year, 2 years, 5 years, and every 5 years thereafter 10. Patients were considered 'lost to follow-up' when the last contact (outpatient clinic or telephone call) was 5 years overdue 11. For patients without any revision, the date of last contact was used for analysis.

### Outcome and endpoints

The primary outcome was defined as implant failure over time after presumably successful DAIR (DAIR cohort) or after implantation of primary or revision arthroplasty not exposed to DAIR (control cohort). Implant survivorship was evaluated with a 2-step approach.

(a) *Clinical failure*: In the first step, the outcome included clinical implant failure requiring revision of the arthroplasty. Implant failure was categorized in septic or aseptic failure. Two endpoints were defined: (i) revision for any reason and (ii) revision for aseptic loosening of any component. For each endpoint, the cumulative incidence was calculated.

(b) *Radiological signs for implant failure*: In the second step, radiographic evidence of loosening of any component was evaluated. At each follow-up, a set of radiographs was obtained: an anterior-posterior pelvic view centered on the symphysis and a false-profile view 12. For radiological analysis, the first postoperative radiograph and the most recent radiograph were compared. The study design for radiological analysis is illustrated in the Supplementary Material (**Figure S2**). All images were corrected for magnification by using the true size of the femoral head. In the case of revision arthroplasty, the radiograph prior to the intervention was analyzed. All radiographs were analyzed for this study in a randomized fashion and blinded as to whether the image was derived from a DAIR or a control cohort. Radiological changes were rated according to the Gruen zones 13 for the stem and DeLee and Charnley zones^14^ for the cup. Radiographs were analyzed for osteolysis, femoral osteomyelitis 15, debonding, stem subsidence (in millimeters), and fracture of the cement mantel 10-12, 16-21. Radiographic evidence of loosening of any component was defined as circumferential osteolysis/debonding around the stem or cup 10-12, 16-21 or subsidence of ≥ 5 mm 19, 22 and/or fracture of the cement mantle 11, 23.

Outcome analysis was performed for the composite cohorts, and for primary and revision THAs separately.

### Statistical methods, sample size, and case matching

We postulated that there would be stable implants in 95% of the THAs in the control cohort 24 and in 75% of the THAs in the DAIR cohort at follow-ups ≥ 5 years 25. The sample size calculation that compared percentages (2-sided, alpha 5%, 80% power to reject the null hypothesis) estimated 49 samples in each arm. Considering that multiple variables may influence implant survivorship, the control cohort was expanded 3-fold via 1:3 matching. DAIR cases and controls were matched for patient age at the time of surgery (younger than 55 years, 55-65 years, 65-75 years, and older than 75 years), sex, type of surgery (primary or revision THA), and stem type. Thus, the study required ≥49 samples in the DAIR cohort and we aimed for ≥147 matched samples in the control cohort to reject the hypothesis.

Considering the long study period, we estimated a mortality rate of 30% in our study population 11, 26. Therefore, a competing risk analysis was included in the statistical plan.

IBM SPSS Statistics (version 24, 2018) and R project version 3.4.3 (2018) were used for statistical analysis. The statistical level of significance was defined as a p-value of < 0.05.

The local ethical committee approved the study protocol (EKNZ No. 2018-01861).

## Results

### Study population for the evaluation of clinical failure

In the study period, we identified 62 THAs exposed to DAIR with a curative intention because of PJI. Five cases were excluded because of missing radiographs (3 hips) and unmatchable prosthetic stems (2 hips). Hence, 56 (29 female and 27 male) patients with DAIR in 57 THAs were included in the DAIR cohort. Except for 1 male patient with infection of both THAs, all patients had a 1-sided PJI. The causative microorganisms are listed in the Supplementary Material (**Table S1**). Given the 1:3 matching, 171 THAs were selected for the control cohort. One subject was lost to follow-up after surgery, and no equivalent control with the same degree of matching variables was found in the arthroplasty register. Thus, 167 (86 female and 81 male) patients with 170 THAs were included in the control cohort. Both study populations were normally distributed (Shapiro-Wilk test). Patient characteristics and THA-associated variables are shown in **Table 1**.

The proportion of primary and revision THAs were similar in both cohorts. In the DAIR cohort, 37 (65%) THAs were primary and 20 (35%) revision implants. In the control cohort, 110 (65%) THAs were primary and 60 (35%) were revision implants.

### Study population for the radiological evaluation of failure

The radiological analysis included a set of images for 56 THAs in the DAIR cohort and a set of images for 168 THAs in the control cohort. In comparison to the study population for clinical failure, 1 THA in the DAIR cohort and 2 THAs in the control cohort were excluded from this analysis because of poor image quality or loss of follow-up images. Thus, the study population included 224 (99%) of 227 THAs within the study population.

### Follow-up period

The mean follow-up periods for all THAs in the DAIR and control cohorts were 6.1 (SD 4.7) and 7.8 (SD 5.5) years, respectively. In the DAIR cohort, the mean follow-up periods for primary and revision THAs were 5.9 (SD 4.7) and 6.4 (SD 4.8) years, respectively. In the control cohort, the mean follow-up periods for primary and revision THAs were 6.7 (SD 5.2) and 9.8 (SD 5.5) years, respectively.

During the entire follow-up period, 20 (36%) of 56 patients in the DAIR cohort died after a mean time of 4.1 (SD 4.7) years, and 54 (32%) of 167 patients in the control cohort died after a mean time of 7.2 (SD 5.4) years. The mean follow-up period for living patients at study termination was 7.2 (SD 4.4) years in the DAIR cohort and 8.1 (SD 5.6) years in the control cohort. In these subgroups, the mean follow-up periods for living patients for primary and revision THAs were 6.5 (SD 4.1) and 8.4 (4.7) years in the DAIR cohort, respectively, and 7.0 (4.9) and 10.4 (6.0) years in the control cohort, respectively.

The difference in proportion of deceased patients in the case and control groups was not statistically significant (p = 0.76). There was no significant difference in the cumulative incidence for death (adjusted for stem revision), when we compared the results in the DAIR cohort with those in the control cohort (p= 0.62). Competing risk analysis showed no significant difference in endpoints when we compared deceased and living patients (Supplementary Material**Figures S5** and** S6**).

### Outcome: Clinical failure

**Figure 1:**
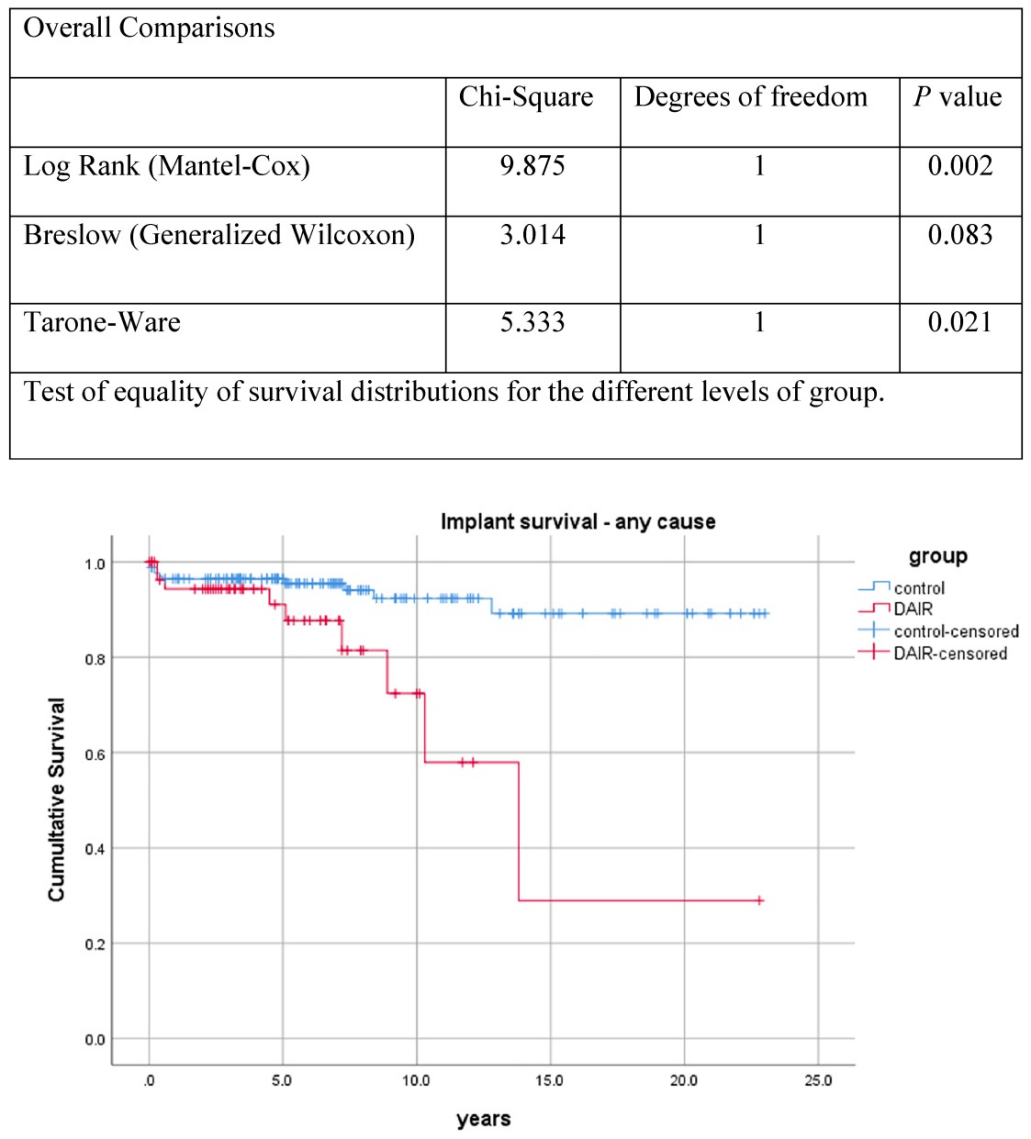
Kaplan-Meier curve of implant survival (revision for any reason). Y-axis, cumulative proportion; X-axis, follow-up in years.

*(i) Endpoint revision for any reason*: Revision of at least 1 component was performed in 9 (16%) THAs in the DAIR cohort and 10 (6%) THAs in the control cohort (p = 0.03). The Kaplan-Meier curve of implant survival of all THAs is illustrated in **Figure 1**. Eight THAs in each cohort had an exchange of the stem during the follow-up period (i.e.; 14% in the DAIR cohort and 5% in the control cohort; p = 0.02) Supplementary material **Figures S3** and **S4**]. The cup was exchanged alongside the stem revision in 6 of 8 cases in the DAIR cohort and in 4 of 8 cases in the control cohort. In addition, isolated cup revision was performed in 1 THA in the DAIR cohort and in 2 THAs in the control cohort.

The reasons for the 9 implant failures in the DAIR cohort were septic failure in 4 (7%) and aseptic failure in 5 (9%) THAs. Septic failure occurred after 3.7, 4.0, 7.5, and 86.2 (or 7.2 years) months after the DAIR procedure. Whereas the first 3 THAs were classified as persistent or relapsing PJIs, the fourth was considered a new hematogenous PJI. The proportion of infection cure in the DAIR cohort was 93%, consistent with our previous study [5. However, the purpose of the study was to analyze implant failure and not infection treatment concept.

The reasons for the 10 implant failures in the control cohort were septic failure in 2 (1%) and aseptic in 8 (5%) THAs. Septic failure occurred after 0.5 and 6.1 months, respectively, after surgery.

The difference in comparison to the DAIR cohort was significant for septic failure (1% versus 7%, p = 0.04) but not for aseptic failure (5% versus 9%, p = 0.32).

*Primary versus revision THAs*: Three of the 8 failed stems in the DAIR cohort were primary THAs (3/37; 8%) and 5 (5/20; 25%) of them were revision THAs. In the control cohort, 6 failed stems were primary THAs (6/110; 5.5%) and 2 (2/60; 3%) of them were revision THAs (p = 0.8). The Kaplan-Meier curves of implant survival (revision of any component for any reason) is separately illustrated for primary and revision THAs in Supplementary Material **Figures S7 and S8,** respectively. The cumulative implant survival was statistically not significantly different in the groups with primary THAs exposed to DAIR and not exposed to DAIR (**Figure S7**). However, Revision THAs exposed to DAIR had a higher failure rate of failure for any reason than did the control cohort (Log Rank Test, p = 0.004; **Figure S8**).

**Figure 2:**
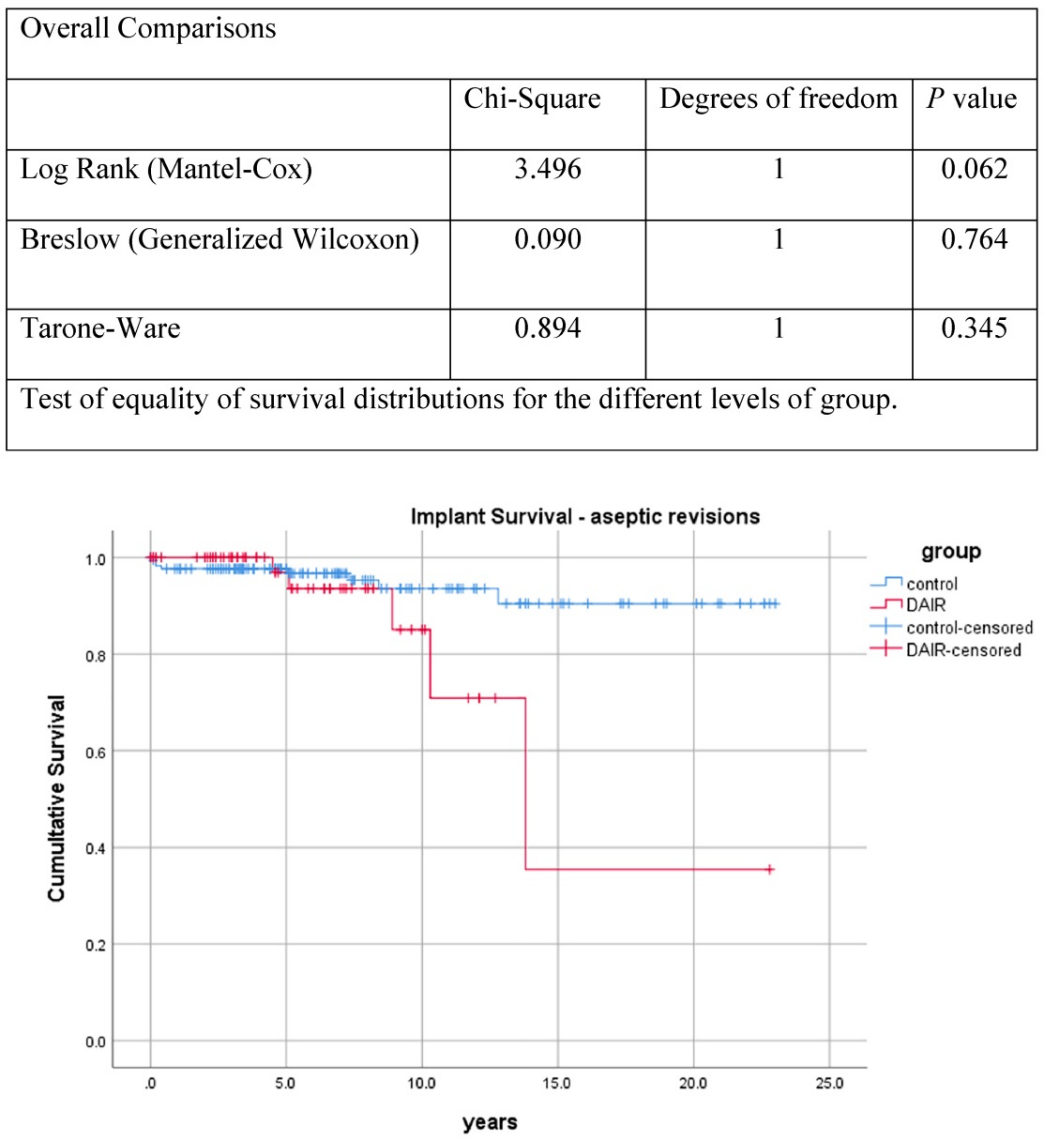
Kaplan-Meier curve of implant survival (revision for aseptic loosening of any component). Y-axis, cumulative proportion; X-axis, follow-up in years.

* (ii) Endpoint “revision for aseptic loosening of any component*”: Aseptic failure occurred 53.9 - 165.0 months after DAIR in the DAIR cohort and 0.7 - 153.2 months in the control cohort (**Table 2**). The time points and detailed reasons for aseptic failure are shown in **Table 2**. The Kaplan-Meier curve of implant survival and revision for aseptic loosening of any component is shown in **Figure 2**. No statistical difference was observed between the DAIR cohort and control cohort. When analyzing primary and revision THAs separately (Table 2 and Kaplan-Meier curve of implant survival in Supplementary **Figure S9** and** S10**), no statistical difference was observed.

### Outcome: Radiological evaluation of failure

Osteolysis or debonding around the stem was evident in 2 (4%) hips in the DAIR cohort and in 3 (2%) in the control cohort (p = 0.60). These 5 hips consisted of 4 cemented straight stems and 1 revision stem. Osteolysis around the cup was detected in 2 (4%) hips in the DAIR cohort and in 3 (2%) in the control cohort (p = 0.60). Stem subsidence of 5 mm or more was seen in 2 (4%) THAs in the DAIR cohort and in 7 (4%) in the control cohort (p = 0.84). The 2 hips in the DAIR cohort consisted of cemented straight stems, and the 7 hips in the control cohort included 2 cemented straight stems, 1 cemented TwinSys stem, and 4 revision stems. A broken cement mantle occurred in 1 subject of each cohort; both stems were cemented straight stems.

Radiographic evidence of loosening of any component according to definition was found in 4 ([7%], 2 stems, 2 cups) of 56 THAs in the DAIR cohort and 8 ([5%], 2 THAs loose on both components, 5 stems, 1 cup) of 168 THAs in the control cohort (p = 1). Of these, 3 of 4 hips in the DAIR cohort and 4 of 8 hips in the control cohort were revised during follow-up. Patients with loose components without revision claimed of no symptoms. The detailed listing of these findings categorized in primary and revision THAs is illustrated in the Supplementary Material (page 12).

## Discussion

Curative surgical strategies for PJI that preserve a functional joint consist of DAIR and 1- or 2-stage exchange of the arthroplasty 4. Although implant stability after 1- and 2-stage exchanges in cured cases has been shown to be excellent 19, 27, this is the first study to investigate implant stability over time *after* DAIR and presumably cure of infection. Consistent with our hypothesis, although in a lower proportion than postulated, we found a higher revision rate of any component for any reason in the DAIR cohort (16%) in comparison to the control cohort (6%). The significance of this difference derives mainly from septic failure cases (7% in the DAIR cohort versus 1% in the control cohort, p = 0.04). In the subgroup analysis of primary and revision THAs, a statistical significant difference of implant failure was seen in revision THAs exposed to DAIR in comparison to the control cohort of revision THAs (Supplementary Material Figure S8).

Our hypothesis for aseptic revisions for any reason (9% versus 5%) and for radiological evidence of loosening (7% versus 5%) was refuted because the differences of 4% and 2%, respectively, were statistically not significant. The implant stability results in the aseptic cases of the control cohort (95%) are comparable to those published in other registries 6-8, 24 and manuscripts that reported long-term follow-ups of patients managed with THA 11, 17, 19-21, 27. The implant stability of the cured PJI cases in our DAIR cohort was demonstrated with a mean follow-up of 6.1 (SD 4.7) years for all hips and 7.2 (SD 4.4) years for hips in living patients. Although the implant stability results in the DAIR cohort were lower than those in the control cohort, our findings are important for clinical decision making. They allow us to assess the clinical relevance of the theoretical aspects of inflammation pathogenesis and of bone and soft tissue damage caused by surgical intervention at the infection site.

In early postoperative PJI, fixation of the THA might be substantially disturbed due to dislocation of the hip and exchange of the mobile parts (e.g., femoral head and liner of the cup) 28. The ingrowth of cementless components in the postoperative period is important for long-term stability of the implant 29. Vigorous manipulation of cemented implants might damage the cement-implant interface, with unfavorable consequences for implant survival 30. In late PJI (i.e.; hematogenous pathogenesis), implant stability can be compromised because of bone tissue damage 31. Excessive removal of infected scar tissue can further compromise the stability of the hip, leading to increased dislocations 32. Conversely, our DAIR cohort demonstrated a high rate of implant stability in aseptic cases.

Our study has limitations. In a double-cohort study, there is potential bias as a result of sampling 2 populations. In the DAIR cohort, all THAs in our institution were included. We further limited this bias by matching the control cohort with numerous previously published risk factors for implant loosening (**Table 1**). We also counterbalanced the heterogeneity of implants with corresponding matching. Data collection in our cohort was prospective, although the analysis for this study was performed retrospectively, leading to less control over measurements. Therefore, all radiographs were reanalyzed for this study in a blinded fashion. Because this was an observational study, there is the possibility of unrecognized confounders. Matching for additional factors typically associated with infection (diabetes mellitus, smoking, rheumatoid arthritis, body mass index and co-morbidity index) was not performed, because this would have further limited the sample size number. The same reason applies to the number of primary and revision cases. The life-time history of previously cured PJI was not assessed patients with revision arthroplasties. However, at the time of study inclusion, none of the subject in the control group had an infection. Analytics for the cause of failure was performed and would have recognized an uneven distribution of failed implants because of infection. The time span of the study (26 years) may add potential confounders in pre-, intra- and postoperative period (e.g.; surgical technique). This is a single-center study, limiting the variability of surgical techniques to a few surgeons specialized in septic surgery 19. Many of the protocols were applied rigidly for over two decades. In retrospect, and in light of the 4% failure difference between aseptic cases in the DAIR and the control cohort, the sample size is relatively small. The detailed case analysis of all included THAs, including competing risk analysis, indicates that our results are valid. Our results are not yet generalizable and need to be confirmed by other centers and registries with larger cohorts and longer follow-up results.

In conclusion, THAs exposed to DAIR were more frequently revised for any reason than were THAs in the control cohort. The significance of this difference derives mainly from septic failure cases. The difference in revisions for aseptic loosening and in radiographic evidence of loosening of any component between the cohorts was not significant. Provided that our study results are confirmed in future studies with a higher sample size and fewer limitations, these data indicate that concerns for long-term implant stability are not justified in the decision making for or against DAIR in hip PJI.

## Supplementary Material

Supplementary figures and tables.

## Figures and Tables

**Table 1:** Patient characteristics and THA-associated variables in the study population*

Characteristics		DAIR Cohort		Control Cohort		Total	
		N	%	N	%	N	%
OP age (yr)	Mean age	71.5		71.7		71.7	
	<55	4	7.0	15	8.8	19	8.3
	55-65	9	15.8	26	15.2	35	15.4
	65-75	18	31.6	54	31.6	72	31.6
	>75	26	45.6	76	44.4	102	44.7
Sex	Female	29	50.9	88	51.5	117	51.3
	Male	28	49.1	83	48.5	111	48.7
Surgery type	Primary	37	64.9	111	64.9	148	64.9
	Revision	20	35.1	60	35.1	80	35.1
Stem type	Cemented straight stems†	16	28.1	49	28.7	65	28.5
	Cemented Twinsys‡	16	28.1	47	27.5	63	27.6
	Uncemented Twinsys‡	11	19.3	33	19.3	44	19.3
	Optimys‡	1	1.8	3	1.8	4	1.8
	Revision stems§	13	22.8	39	22.9	52	22.9

*DAIR = debridement and implant retention; THA = total hip arthroplasty; OP age = patient age at the time point of surgery. †Müller type straight stems + Virtec straight stems (both Zimmer, Winterthur, Switzerland). ‡Mathys, Bettlach, Switzerland. §Revitan + Wagner SL revision stems (both Zimmer, Winterthur, Switzerland); Centris stem (Mathys, Bettlach, Switzerland).

**Table 2:** Aseptic failures in the DAIR and control cohorts^*^

Primary THAs					
**DAIR cohort**			**Control cohort**		
Months after DAIR	Surgery	Indication	Months after implantation	Surgery	Indication
53.9	Exchange of THA	Pain, stem loosening	0.7	Stem exchange	Dislocation with failed relocation
106.3	Stem exchange	Pain, stem loosening	2.2	Cup exchange	Recurrent dislocation
			87.8	Exchange of THA	Squeaking hip, stem loosening
**Revision THAs**					
**DAIR cohort**			**Control cohort**		
61.3	Cup exchange	Loosening of the cup	1.8	Cup exchange	Recurrent dislocation
123.3	Stem exchange	Pain, stem loosening	5.1	Stem exchange	Stem subsidence, gluteal insufficiency
165.0	Exchange of THA	Pain, stem loosening	60.9	Exchange of THA	Pain, stem loosening
			100.9	Stem exchange	Pain, stem loosening
			153.2	Stem exchange	Pain, stem loosening

*DAIR = debridement and implant retention; THA = total hip arthroplasty.
