# Supplementary material for: Debridement, Antibiotics and Implant Retention for Hip Periprosthetic Joint Infection: Analysis of Implant Survival after Cure of Infection
Source: J Bone Jt Infect. 2020 Feb 10;5(1):35–42. doi: 10.7150/jbji.40924 (PMC7045527; doi:10.7150/jbji.40924)
Supplement: Supplementary file 1 — Supplementary figures and tables. [file jbjiv05p0035s1.pdf]

Supplementary Material **Page**

The study design for clinical failure analysis (**Figure S1**) 2

The study design for radiological analysis (**Figure S2**) 3

The causative microorganisms (**Table S1**) 4

Flow chart of included THAs, proportion of unrevised and revised stems, and the distribution of living and deceased patients in the DAIR cohort (**Figure S3**) 5

Flow chart of included THAs, proportion of unrevised and revised stems, and the distribution of living and deceased patients in the control cohort (**Figure S4**) 5

Competing risk analysis (implant failure versus death). Cumulative Incidence of Implant failure. Revision of any component for any reason (**Figure S5**) 6

Competing risk analysis (implant failure versus death). Cumulative Incidence of Implant failure. Revision for any component for aseptic loosening (**Figure S6**) 7

Kaplan-Meier curve of implant survival **primary** THAs (revision for any reason). (**Figure S7**) 8

Kaplan-Meier curve of implant survival **revision** THAs (revision for any reason). (**Figure S8**) 9

Kaplan-Meier curve of implant survival **primary** THAs (revision for aseptic loosening). (**Figure S9**) 10

Kaplan-Meier curve of implant survival **revision** THAs (revision for aseptic loosening). (**Figure S10**) 11

Outcome: Radiological evaluation of failure, findings categorized in primary and revision THA 12

Figure S1. The study design for clinical failure analysis.

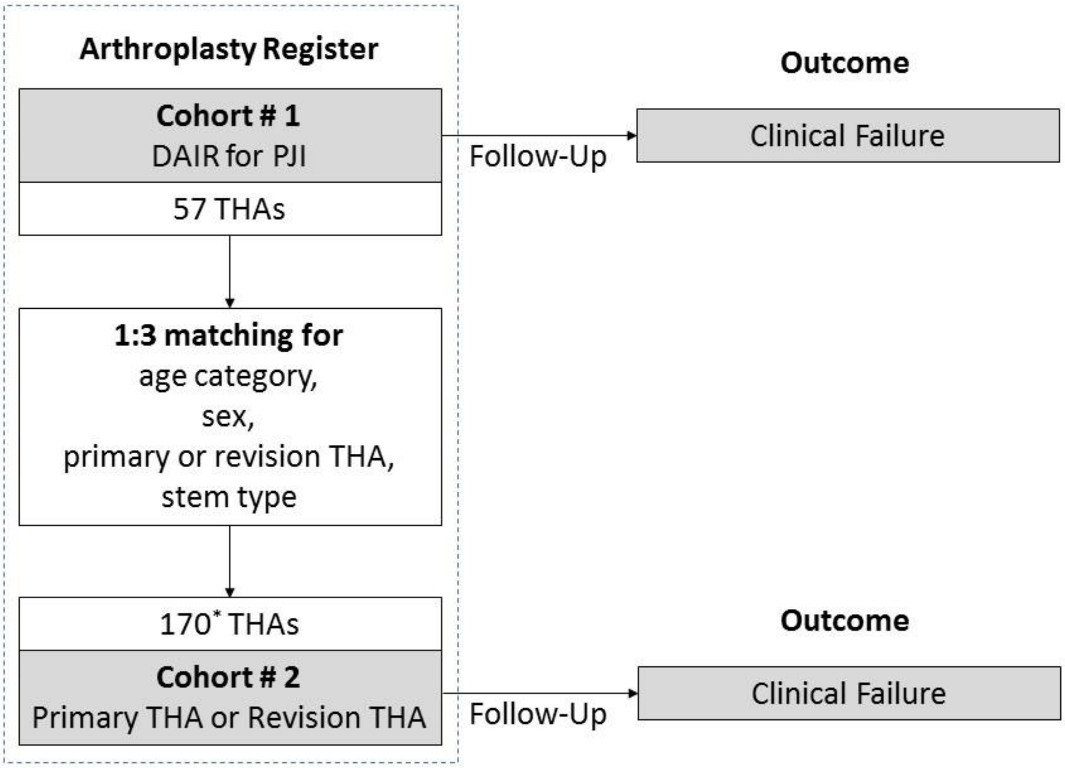

* 171 THAs were selected for the control cohort. One subject was lost to follow-up after surgery, and no equivalent control with the same degree of matching variables was found in the arthroplasty register.

Figure S2. The study design for radiological analysis.

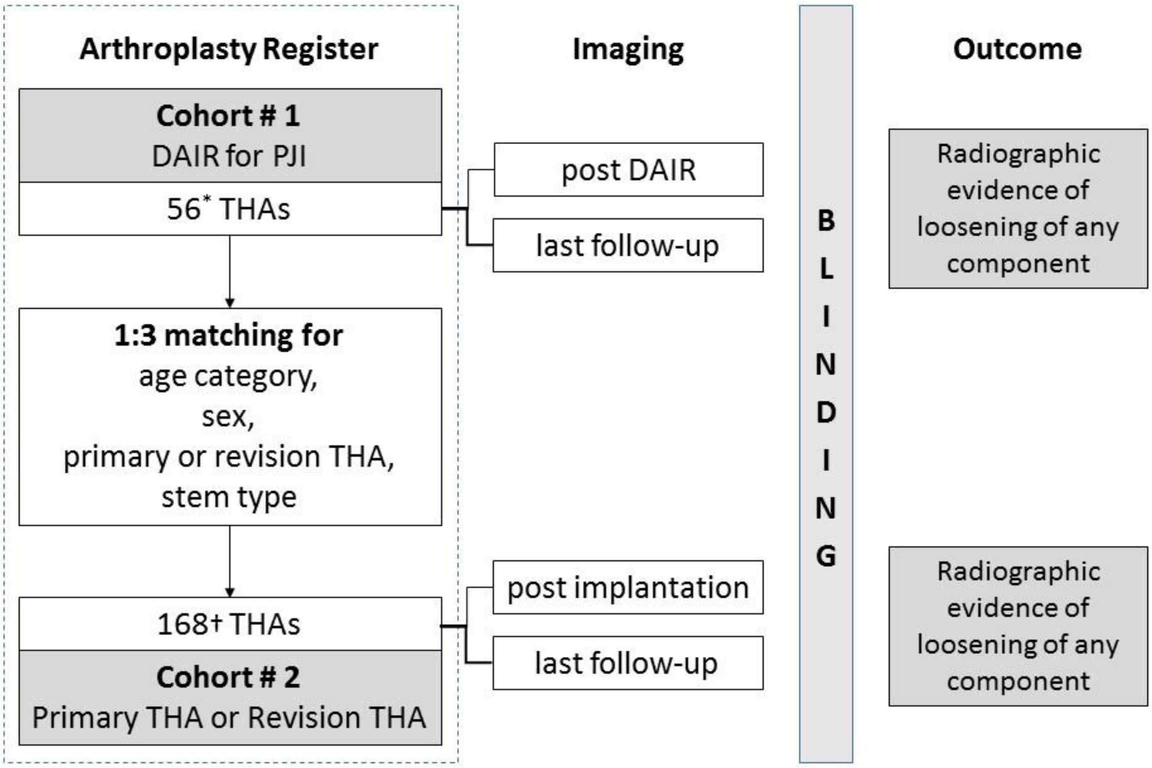

The radiological analysis included a set of images for 56 THAs in the DAIR cohort and a set of images for 168 THAs in the control cohort.

In comparison to the study population for clinical failure, *1 THA in the DAIR cohort and †2 THAs in the control cohort were excluded from this analysis because of poor image quality or loss of follow-up images.

All radiographs were re-analyzed for this study in a randomized fashion and blinded as to whether the image was derived from a DAIR or a control cohort.

Table S1. The causative microorganisms in 57 PJIs and treated with DAIR.

**Microorganisms**	**N**
**Monomicrobial infections**	43 (75)
Coagulase-negative staphylococci	21 (11)
Gram-negative bacilli*	21 (11)
Staphylococcus aureus	16 (14)
Streptococcus spp.	8 (6)
Enterococcus spp.	4 (7)
Anaerobes†	5 (1)
Aerococcus viridans	1
**Polymicrobial infections†**	13 (23)
**Culture-negative PJI**	1

†In polymicrobial infections, 2 microorganisms were involved in 8 PJIs, and 3 in 3 PJIs, 4 in 2 PJIs.

*Gram-negative bacilli included *Escherichia coli* (9), *Enterobacter* spp. (4), *Pseudomonas aeruginosa* (2), *Proteus mirabilis* (2), *Acinetobacter Iwoffii* (1) *Citrobacter koserii* (1), *Morganella morganii (1)*, *Klebsiella oxytoca* (1).

†Anaerobes included *Cutibacterium* spp. (2), *Actinomyces* spp. (1), *Bacillus* spp. (1), *Finegoldia magna* (1).

Figure S3. Flow chart of included THAs, proportion of unrevised and revised stems, and the distribution of living and deceased patients in the DAIR cohort.

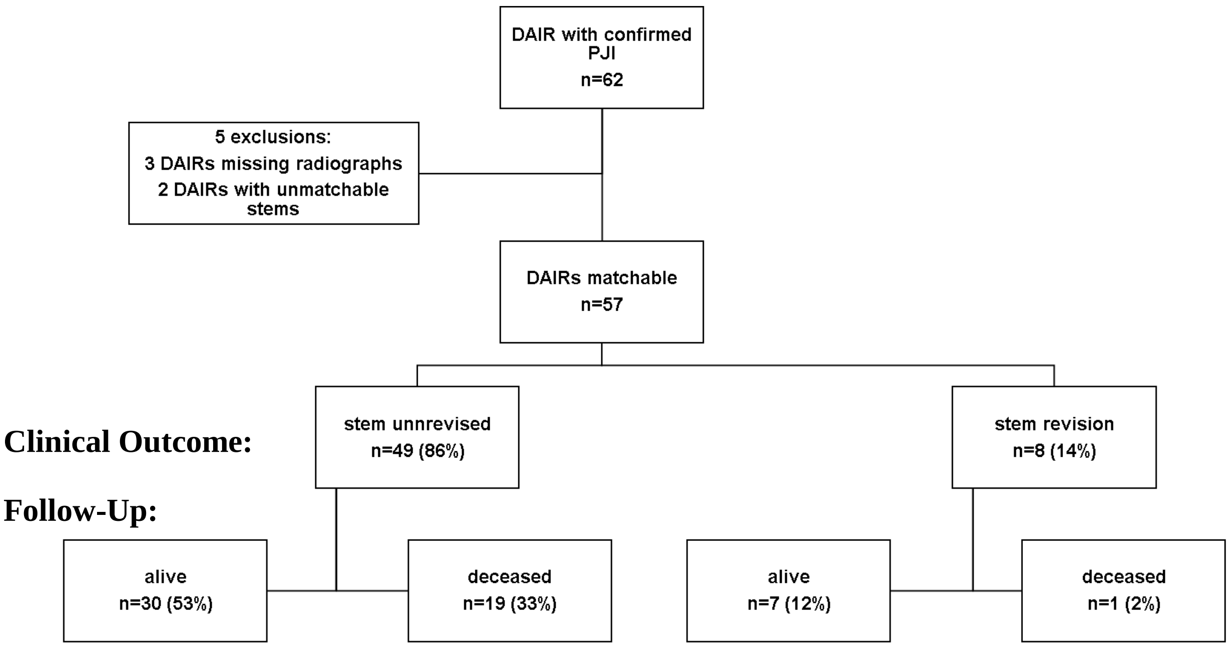

Figure S4. Flow chart of included THAs, proportion of unrevised and revised stems, and the distribution of living and deceased patients in the control cohort.

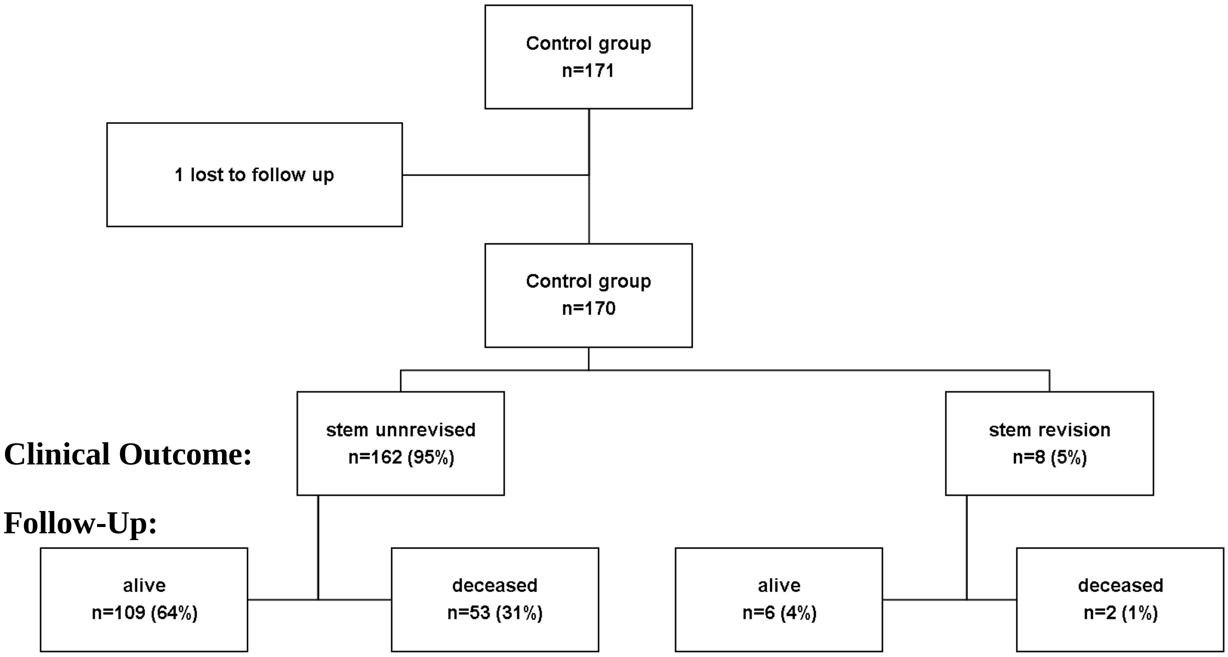

Figure S5. Competing risk analysis (implant failure versus death). Cumulative Incidence of Implant failure. Revision of any component for any reason.

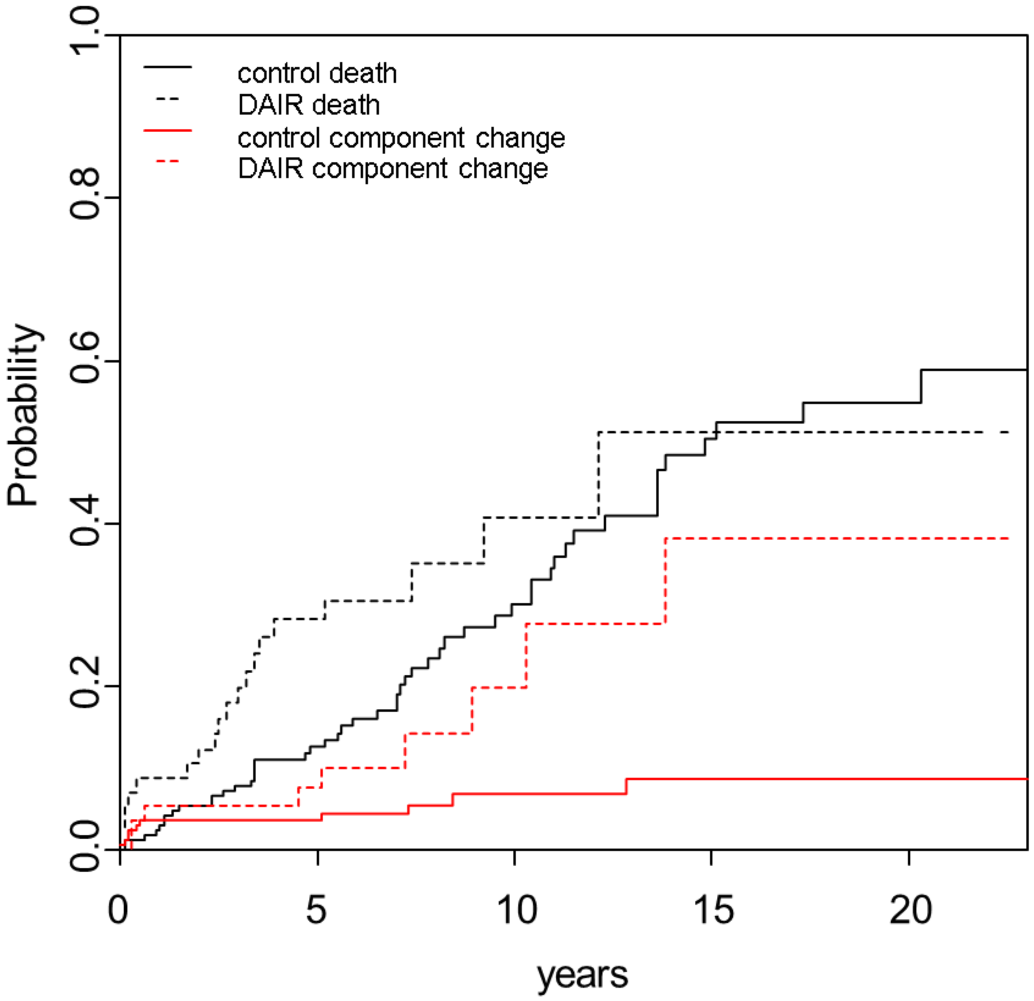

Any component any cause					
Test equality across groups (Gray):					
		p-value			
1: Death		0.1475029			
2: Component change		0.0064984			
estimates at time points					
years	0	5	10	15	20
control 1	0	0.12568	0.3021	0.50506	0.54987
DAIR 1	0.017544	0.28244	0.40844	0.51338	0.51338
control 2	0.005882	0.03529	0.06722	0.08526	0.08526
DAIR 2	0	0.07556	0.19802	0.38167	0.38167
Standard errors:					
years	0	5	10	15	20
control 1	0	0.02656	0.04582	0.05999	0.06249
DAIR 1	0.017544	0.06312	0.09003	0.13174	0.13174
control 2	0.005882	0.01419	0.02306	0.02897	0.02897
DAIR 2	0	0.03714	0.07891	0.1454	0.1454

Figure S6. Competing risk analysis (implant failure versus death). Cumulative Incidence of Implant failure. Revision of any component for aseptic loosening.

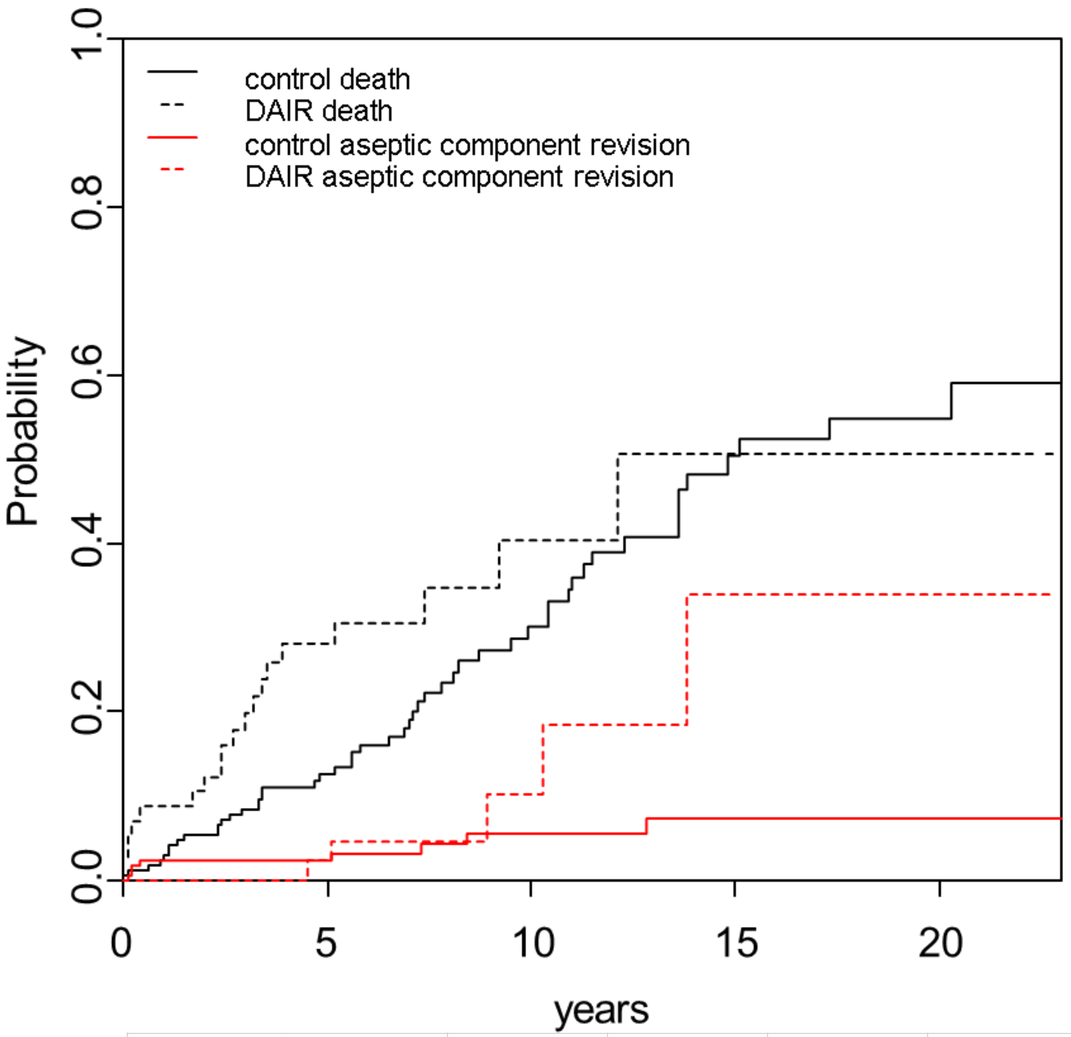

Any component aseptic					
Test equality across groups (Gray):					
		p-value			
1: Death		0.12863			
2: Component change		0.1093			
Estimates at time points:					
years	0	5	10	15	20
control 1	0.005882	0.12538	0.3017	0.50388	0.55012
DAIR 1	0.017544	0.28106	0.4033	0.50648	0.50648
control 2	0	0.02353	0.0555	0.07336	0.07336
DAIR 2	0	0.02247	0.1015	0.33877	0.33877
Standard errors:					
years	0	5	10	15	20
control 1	0.005882	0.02651	0.04579	0.06009	0.06299
DAIR 1	0.017544	0.0628	0.08814	0.12839	0.12839
control 2	0	0.01166	0.02171	0.02783	0.02783
DAIR 2	0	0.02255	0.06331	0.18084	0.18084

Figure S7: Kaplan-Meier curve of implant survival **primary** THAs (revision for any reason). Y-axis, cumulative proportion; X-axis, follow-up in years.

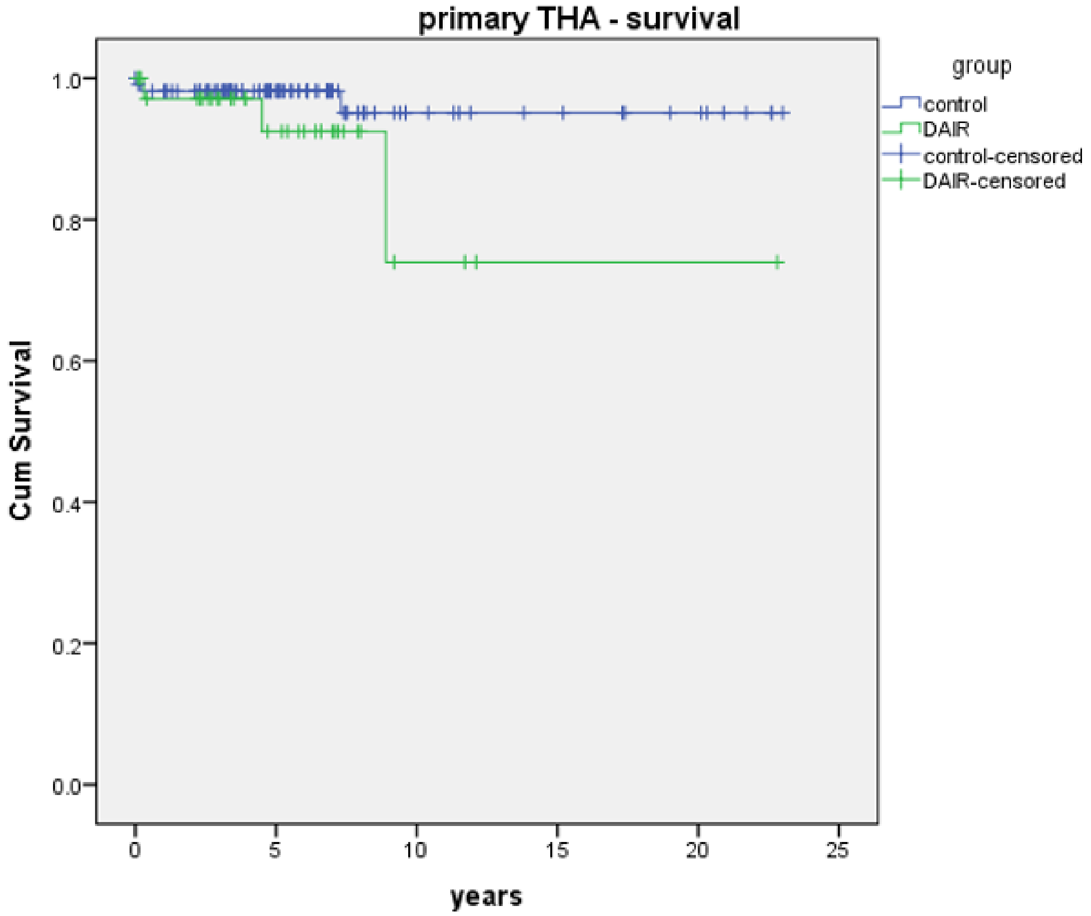

Case Processing Summary

group	Total N	N of Events	Censored	
			N	Percent
control	110	3	107	97.3%
DAIR	37	3	34	91.9%
Overall	147	6	141	95.9%

Overall Comparisons

	Chi-Square	df	Sig.
Log Rank (Mantel-Cox)	2.539	1	.111
Breslow (Generalized Wilcoxon)	1.036	1	.309
Tarone-Ware	1.626	1	.202

The vector of trend weights is -1, 1. This is the default.

Figure S8: Kaplan-Meier curve of implant survival **revision** THAs (revision for any reason). Y-axis, cumulative proportion; X-axis, follow-up in years.

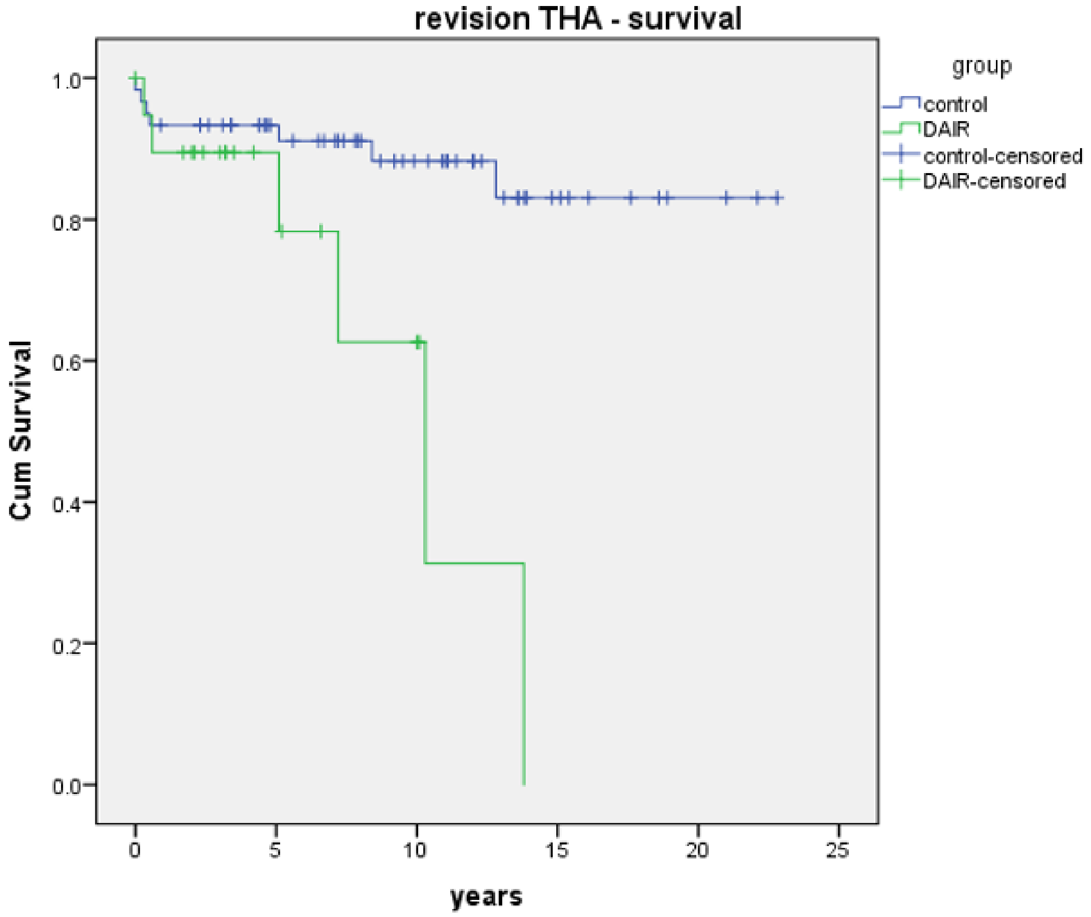

Case Processing Summary

group	Total N	N of Events	Censored	
			N	Percent
control	60	7	53	88.3%
DAIR	20	6	14	70.0%
Overall	80	13	67	83.8%

Overall Comparisons

	Chi-Square	df	Sig.
Log Rank (Mantel-Cox)	8.135	1	.004
Breslow (Generalized Wilcoxon)	2.707	1	.100
Tarone-Ware	4.598	1	.032

The vector of trend weights is -1, 1. This is the default.

Figure S9: Kaplan-Meier curve of implant survival **primary** THAs (revision for aseptic loosening). Y-axis, cumulative proportion; X-axis, follow-up in years.

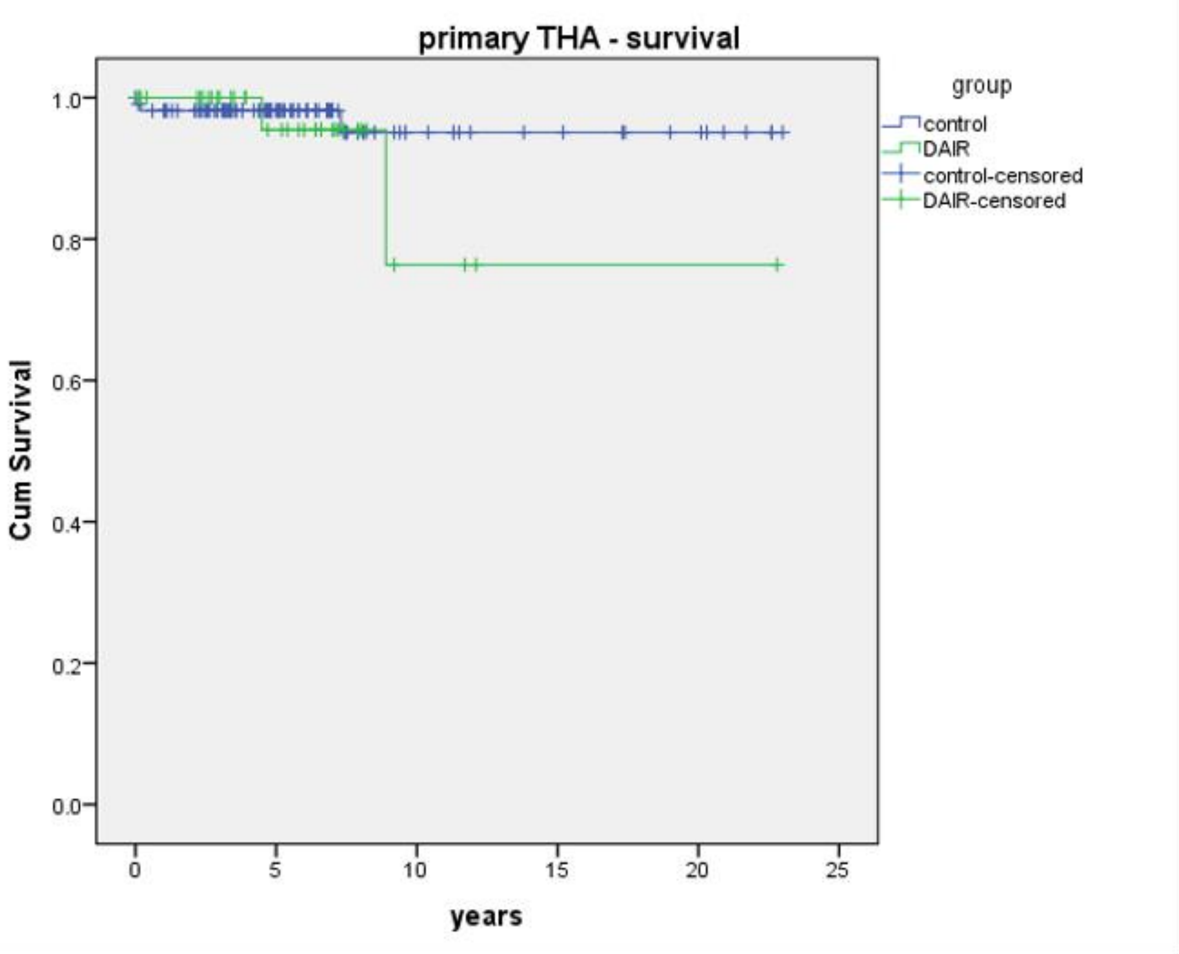

Case Processing Summary

group	Total N	N of Events	Censored N	Censored Percent
control	110	3	107	97.3%
DAIR	37	2	35	94.6%
Overall	147	5	142	96.6%

Overall Comparisons

	Chi-Square	df	Sig.
Log Rank (Mantel-Cox)	.816	1	.366
Breslow (Generalized Wilcoxon)	.010	1	.919
Tarone-Ware	.197	1	.657

The vector of trend weights is -1, 1. This is the default.

Figure S10: Kaplan-Meier curve of implant survival **revision** THAs (revision for aseptic loosening). Y-axis, cumulative proportion; X-axis, follow-up in years.

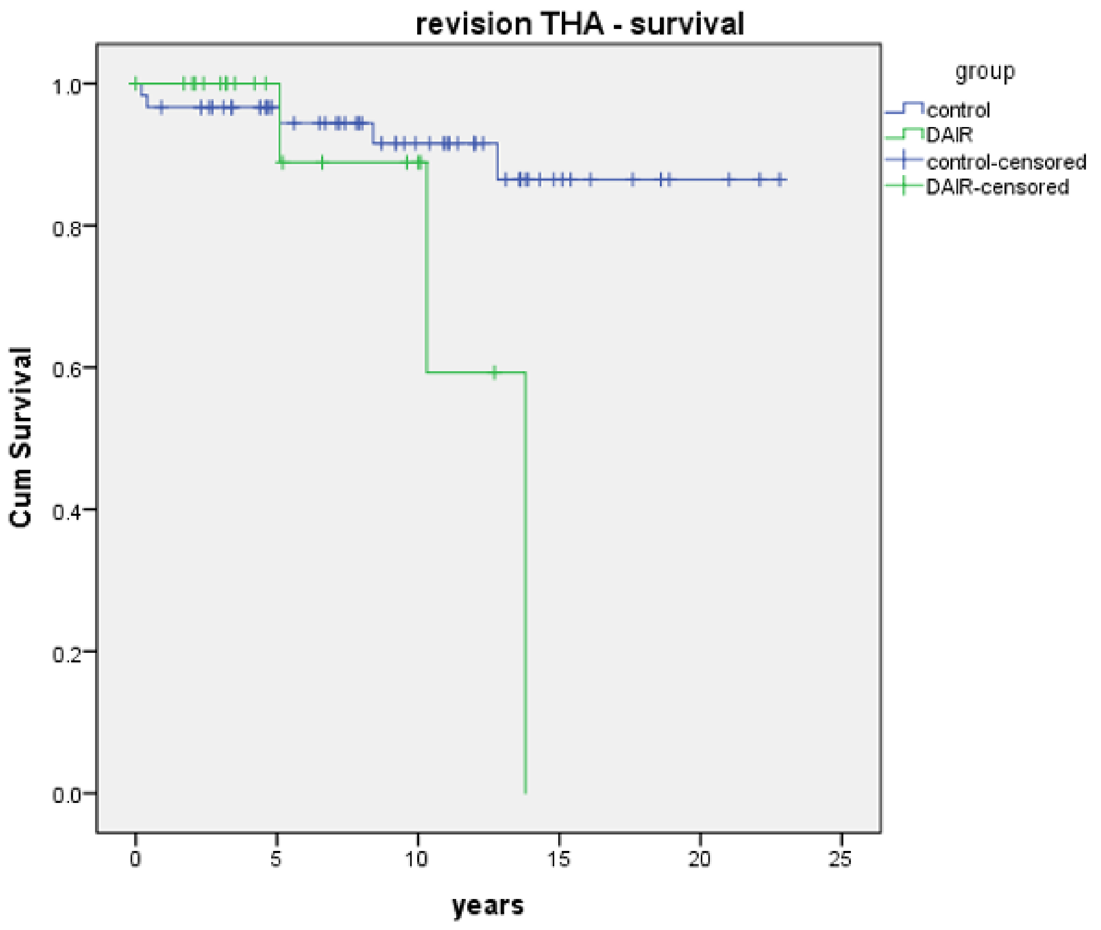

Case Processing Summary

group	Total N	N of Events	Censored	
			N	Percent
control	60	5	55	91.7%
DAIR	20	3	17	85.0%
Overall	80	8	72	90.0%

Overall Comparisons

	Chi-Square	df	Sig.
Log Rank (Mantel-Cox)	3.263	1	.071
Breslow (Generalized Wilcoxon)	.253	1	.615
Tarone-Ware	1.100	1	.294

The vector of trend weights is -1, 1. This is the default.

***Outcome: Radiological evaluation of failure,* findings categorized in primary and revision THAs.**

1 THA (1 revision) in the DAIR cohort and 2 THAs (2 primary) in the control cohort were excluded from this analysis because of poor image quality or loss of follow-up images. Thus, the study population included 224 (99%) of 227 THAs within the study population.

Included:
DAIR cohort 56 THAs: 37 (66%) primary, 19 (34%) revisions THAs
Control cohort 168 THAs: 108 (64%) primary, 60 (36%) revisions THAs

Osteolysis or debonding around the stem

- DAIR cohort: 2 (4%) hips; (1 (3%) primary THA, 1 (5%) revision THA).
- Control cohort in in 3 (2%) hips; (1 (1%) primary THA, 2 (3%) revision THAs).

Osteolysis around the cup

- DAIR cohort: 2 (4%) hips; (1 (3%) primary THA, 1 (5%) revision THA).
- Control cohort: 3 (2%) hips; (1 (1%) primary THA, 2 (3%) revision THAs).

Stem subsidence of 5 mm or more

- DAIR cohort: 2 (4%) hips; (1 (3%) primary THA, 1 (5%) revision THA).
- Control cohort: 7 (4%) hips; (2 (2%) primary THAs, 5 (8%) revision THAs).

The 2 hips in the DAIR cohort consisted of cemented straight stems (1 primary and 1 revision THA), and the 7 hips in the control cohort included 2 cemented straight stems (1 primary and 1 revision THA), 1 (primary THA) cemented TwinSys stem, and 4 revision stems.

Broken cement mantle

- DAIR cohort: 1 (2%) hip; (revision THA).
- Control cohort: 1 (1%) hip; (revision THA)

Radiographic evidence of loosening of any component

- DAIR cohort: 4 (7%) stems; (2 (5%) primary THAs, 2 (11%) revision THAs).
- DAIR cohort: 2 (4%) cups; (1 primary THA, 1 revision THA).
- Control cohort: 8 (5%) hips loose on both components; (2 (2%) primary THAs, 6 (10%) revision THAs).
- Control cohort: 5 (3%) stems; (1 primary THA, 4 revision THAs).
- Control cohort: 1 (1%) cup; (1 revision THA).
